# Damage Inside Borosilicate Glass by a Single Picosecond Laser Pulse

**DOI:** 10.3390/mi12050553

**Published:** 2021-05-13

**Authors:** Weibo Cheng, Jan-Willem Pieterse, Rongguang Liang

**Affiliations:** 1 Wyant College of Optical Sciences, University of Arizona, 1630 East University Boulevard, Tucson, AZ 85721, USA; rliang@optics.arizona.edu; 2 Lumentum, 1750 Automation Pkwy Num 1873, San Jose, CA 95131, USA; Jan-Willem.Pieterse@lumentum.com

**Keywords:** laser-matter interaction, ultrashort pulse laser processing, plasma dynamics

## Abstract

We investigate damage inside the bulk of borosilicate glass by a single shot of IR picosecond laser pulse both experimentally and numerically. In our experiments, bulk damage of borosilicate glass with aspect ratio of about 1:10 is generated. The shape and size of the damage site are shown to correspond to an electron cloud with density of about 10${}^{20}$ cm${}^{-3}$. The underlying mechanism of electron generation by multiphoton ionization and avalanche ionization is numerically investigated. The multiphoton ionization rate and avalanche ionization rate are determined by fitting experimental results. The relative role of multiphoton ionization and avalanche ionization are numerically studied and the percentage of electron contribution from each ionization channel is determined.

## 1. Introduction

Ultrafast lasers are ubiquitous in a wide range of applications such as micro-welding [1], waveguide writing [2,3], and microfluidics [4]. The mechanism of bulk material modification by ultrafast lasers are still an active research topic [5,6,7,8,9,10,11]. The general physical picture of internal transparent material modification can be described as the following processes. When a high intensity laser pulse is focused inside the bulk material, electrons are excited from the valence band to conduction band through multiphoton ionization and subsequent avalanche ionization at the focal volume. The excited electrons form a plasma cloud and absorb part of the pulse energy, which eventually transfers to the lattice through electron phonon interaction and heats up the bulk of the material. When the material exposed to multiple pulses with pulse to pulse interval less than the thermal diffusion time of the material, the heat will accumulate at the focal region and the temperature at the focal region will increase to a few thousands degrees [12,13]. The internal modification of glass by multiple ultrashort laser pulses with high repetition rate has been demonstrated experimentally [14,15,16,17,18,19] and the shape of the modified structure has been studied numerically [7,15]. Under the irradiation of multiple ultrashort laser pulses, the modification zone in the bulk of glass typically contains an outer elliptical zone with an inner darker damaged center [19]. The mechanism of the outer zone formation has been previously understood as a result of the laser heat impact that accumulated during multiple pulse irradiation [14]. However, the formation mechanism of the inner darker damage center is not fully understood [7]. The fundamental picture of how electrons are generated during the very first pulse of the entire pulse train that led to the inner damage center is still not very clear.

In this paper, we target to gain fundamental insights of how ultrashort pulses interact with bulk glass, particularly how initial electrons are generated at the very beginning of the process. Specifically, we focus on understanding the contribution of electrons from different ionization channels such as multiphoton ionization and avalanche ionization during the exposure of a single picosecond laser pulse focused in the bulk of borosilicate glass. We demonstrate experimentally the bulk modification of borosilicate glass with high aspect ratio damage tracks by a single shot of picosecond laser pulse. We present a beam propagation model that tailored to our experiments to extract ionization constants and electron density distributions. The shape and size of the inner dark damage center match an electron density of about 10${}^{20}$ cm${}^{-3}$ very well. We also analyze the relative role of electron contributions from multiphoton ionization and avalanche ionization.

## 2. Experiments

**Figure 1:**
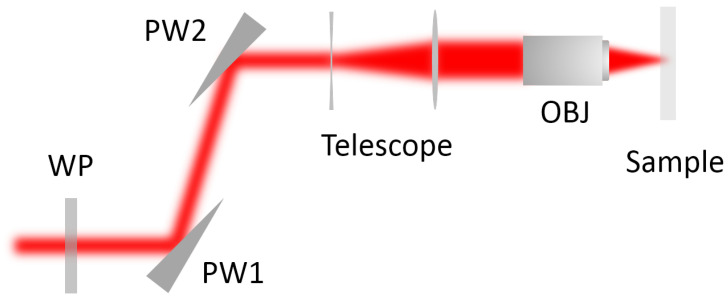
Schematic of the experimental setup. WP: λ/2 plate; PW1, PW2: prism wedge; Telescope: telescope beam expander; OBJ: microscope objective.

The experimental setup used in our study is shown in Figure 1. The laser source is a commercially available Nd:YAG system from Lumentum. It delivers 10 ps pulses at a center wavelength of 1064 nm with a repetition rate up to 8.2 MHz. The maximum available pulse energy from the system is about 400 µJ, which is more than enough for the pule energy that needed in our experiments. The output beam has a close to perfect Gaussian distribution. The analogue power resolution of the laser system is about 0.1% corresponding to pulse energy of about 0.4 µJ. The excessive pulse energy is attenuated by a half-wave plate followed by reflection off a prism wedge mounted close to the Brewster’s angle of the incident beam. This attenuation scheme combines with the analog laser power control enables us to continuously adjust the pulse energy down to sub-microjoule level while maintaining pure polarization state of the attenuated laser beam. More details of this method can be found in a previous study [20]. An infinity-corrected microscope objective with numerical aperture of NA = 0.25 is used to focus the beam into the bulk of borosilicate glass plate. The focal spot size under our focusing geometry is about 5 µm.

The borosilicate glass plate used in our experiments has a thickness of about 1 mm, with density 2.38 $g/{\mathrm{cm}}^{3}$ and refractive index of 1.5 at wavelength 1064 nm. The glass plate is mounted on a linear motorized stage. To ensure single shot exposure, the laser system is operated at a repetition rate of 2 kHz while the stage is translated at a speed of 200 mm/s. The combination effect is a pulse to pulse distance of about 100 µm, which is about 20 times larger than the spot size. The sample and linear motorized stage are mounted on a precision Z-positioning stage that can be accurately adjusted to tune the focal position. Careful adjustment procedure has been implemented to make sure the beam is focused about 100 µm underneath the borosilicate glass surface to generate bulk damage while maintaining minimum spherical aberrations. The pulse energy is carefully measured at the output of the objective with a semiconductor energy sensor. To visualize the damage site, we use a high magnification optical microscope with backside illumination to observe the laser modified region from the side edge of the glass plate.

## 3. Modeling

To understand the mechanism of damage formation, we numerically model the pulse propagation and electron generation inside the borosilicate glass plate. The model used in our study has been widely applied for propagating intense pulse in fused silica [5], air [21], and liquids [22]. We model the linear polarized beam by the electrical field envelop E of the electrical field *E* in cylindrical symmetry coordinates around the propagation axis Z. The electrical field envelop E at the starting position can be expressed by [5],

(1) $$E\left(r,t,0\right)=E_{0}exp\left(-r^{2}/w_{0}^{2}-t^{2}/t_{p}^{2}-ikr^{2}/2f\right)$$

where $E_{0}$ is the amplitude of the initial field, $w_{0}=w_{f}{\left(1+d^{2}/z_{f}^{2}\right)}^{1/2}$ is the beam radius at the start position of the simulation, $w_{f}=2.5$ µm is the beam waist, d is the distance between the focus and the start position, $z_{f}=\pi w_{f}^{2}n_{0}/\lambda_{0}=27.7$ µm is the Rayleigh length, $t_{p}=10$ ps is the FWHM pulse duration, $k=n_{0}\omega_{0}/c$, $n_{0}=1.5$ for borosilicate glass, $\omega_{0}$ is the frequency of the carrier wave, $f=d+z_{f}^{2}/d$ is the curvature of the beam at the starting position.

It is a common practice to use E as an approximation of the electric field *E* and assume the envelop function is slowly varying in both time and space. The evolution of the field envelop can be expressed by [5],

(2) $$\begin{array}{cc} \frac{\partial E}{\partial z}= & \frac{i}{2k}\left(\frac{\partial^{2}}{\partial r^{2}}+\frac{1}{r}\frac{\partial }{\partial r}\right)E-i\frac{k^{\prime \prime }}{2}\frac{\partial^{2}E}{\partial \tau^{2}}+ik_{0}n_{2}{\left|E\right|}^{2}E-\frac{\sigma }{2}\left(1+i\omega_{0}\tau_{c}\right)\rho E-\frac{1}{2}\frac{W_{PI}U_{i}}{{\left|E\right|}^{2}}E \end{array}$$

The propagation equation is described in the reference frame moving at the group velocity $v_{g}{=\partial \omega /\partial k|}_{\omega_{0}}$, $\tau =t-z/v_{g}$. The first term on the right hand side of Equation (2) describes diffraction in the transverse plane. The second term accounts for group velocity dispersion. The third term accounts for Kerr self-focusing with critical power $P_{cr}=\lambda^{2}/2\pi n_{0}n_{2}=3.5$ MW, where $n_{2}=3.45\times 10^{-16}$ ${\mathrm{cm}}^{2}/W$ [7] is the nonlinear part of the refractive index. The fourth term accounts for plasma absorption and plasma defocusing, the cross section for inverse Bremsstrahlung follows the Drude model [5] and the cross section $\sigma =k\omega_{0}\tau_{c}/n_{0}^{2}\rho_{c}\left(1+\omega_{0}^{2}\tau_{c}^{2}\right)=9.7\times 10^{-19}$ ${\mathrm{cm}}^{2}$, where $\tau_{c}=2.33\times 10^{-14}$ s denotes electron collision time, and $\rho_{c}=10^{21}$ ${\mathrm{cm}}^{-3}$ is the critical plasma density at which level the plasma becomes opaque [5]. The fifth term describes the photon ionization of the media by the laser pulse, where $U_{i}=3.7$ eV is the band gap of borosilicate glass.

In our study, the maximum intensity used is $I=2.54\times 10^{12}$ $W/{\mathrm{cm}}^{2}$, which corresponding to the Keldysh parameter $\gamma =\omega_{0}\sqrt{0.64m_{e}U_{i}}/eE=2$ [23]. When γ>1, the multiphoton ionization process will be dominating, while at γ<1 the tunneling ionization process will be dominating. Thus, we approximate the photon ionization rate to the multiphoton ionization rate $W_{PI}=\sigma_{4}I^{4}\rho_{at}$, where $\sigma_{4}$ is the cross section for a four photon process to promote an electron from valence band to conduction band at 1064 nm in borosilicate glass.

The electron excitation by the laser pulse from valence band to conduction band can be described through a rate equation [5],

(3) $$\begin{array}{c} \frac{\partial \rho }{\partial t}=W_{PI}+\eta \rho {\left|E\right|}^{2}-\frac{\rho }{\tau_{r}} \end{array}$$

The first term on the right hand side describes the electron promotion through multiphoton ionization and the second term describes electron generation through avalanche ionization, where $W_{PI}=\sigma_{4}I^{4}\rho_{at}$, $\eta =\sigma /U_{i}$ and $\rho_{at}=2.1\times 10^{22}$ ${\mathrm{cm}}^{-3}$ is the background atom density. The third term represents electron recombination with a characteristics time $\tau_{r}=150$ fs in glass [24].

## 4. Results and Discussion

**Figure 2:**
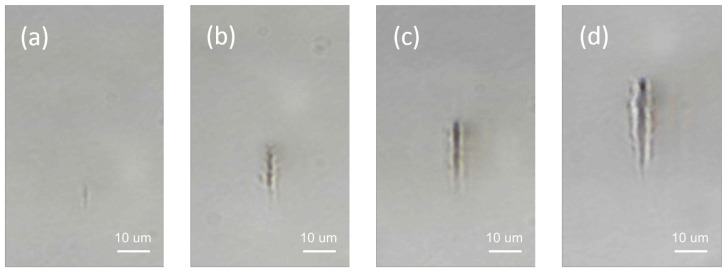
Damage created inside borosilicate glass using single shot of picosecond laser pulse. The focal position is about 100 µm underneath the front surface of the borosilicate glass plate. The laser beam is propagating from top to bottom. The pulse energy is chosen to produce from just visible laser damage to obvious damage tracks inside the glass plate. For the cases shown here, the pulse energy are (**a**) 1 µJ, (**b**) 2 µJ, (**c**) 3 µJ, (**d**) 5 µJ.

The experimental results of damage inside borosilicate glass are shown in Figure 2. For all the cases, we found no surface damages on the glass plate. At around a pulse energy of about 1 µJ, we start observing bulk damages inside the borosilicate glass plate. The length of the damage track increases as we increase the pulse energy from 1 µJ to 5 µJ. The starting point of the damage track shift towards the incoming laser beam as nonlinear effect drives the plasma generation towards the incoming beam when we increase the pulse energy. This phenomenon is also observed in other studies [7,14,15].

**Figure 3:**
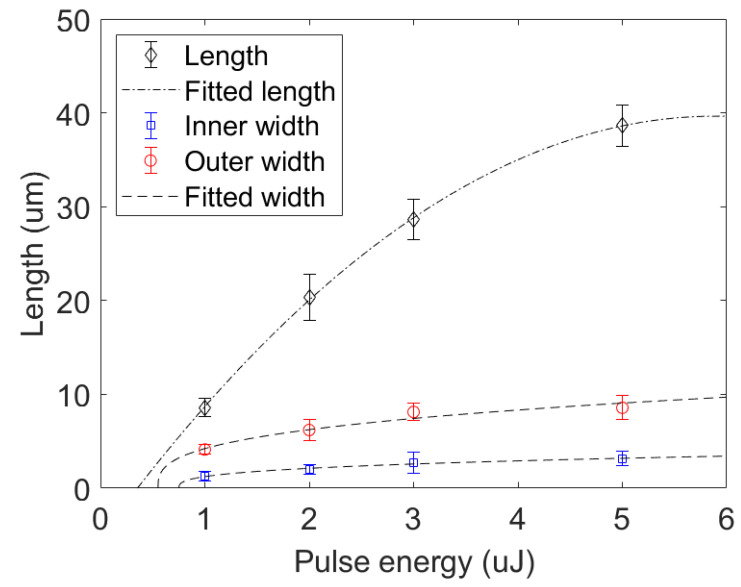
Damage sizes vs. input pulse energy. The length of the damage track vs. the input pulse energy are indicated by the diamond mark. The dotted dashed line is a polynomial fit of the damage length with two degree of freedom. The inner and outer width of the damage track vs. pulse energy are indicated by square and circle mark, respectively. The dashed line is fitted based on Equations (4) and (5).

We measure the length as well as the inner and outer width of the damage track for each of the energy cases shown in Figure 3. At pulse energy of 5 µJ, the damage tracks reach to a length of about 38 µm, which is about 1.4 times that of the Rayleigh length under our focusing geometry. The damage track has an inner width to length aspect ratio of about 1:10 at this energy level. The morphology of the damage track appears to have distinct inner and outer features where the inner damage zone appears to be darker with hollow like shape. Similar morphology has been observed using tightly focused Gaussian beams in fused silica and sapphire [11,25]. The inner and outer width of the damage track can be fitted using the relation [6],

(4) $$D_{inner}=c_{1}\sqrt[{3}]{\left(E_{pulse}-E_{th,i}\right)}$$

(5) $$D_{outer}=c_{2}\sqrt[{3}]{\left(E_{pulse}-E_{th,o}\right)}$$

where $c_{1}$ and $c_{2}$ are fitting constants, $E_{pulse}$ is the input pulse energy, $E_{th,i}$ is the threshold energy for inner damage track, $E_{th,o}$ is the threshold energy for outer damage track. The threshold energy for laser damages in glass is fitted to be about $E_{th,o}=0.55$ µJ, and the threshold energy for generating inner structure is fitted to be about $E_{th,i}=0.75$ µJ. The length of the damage track can be fitted very well by a polynomial function with two degrees of freedom. The polynomial fitted threshold for generating damage is about $E_{th}=0.4$ µJ, which is close to the threshold $E_{th,o}=0.55$ µJ using the relation in Equation (5).

**Figure 4:**
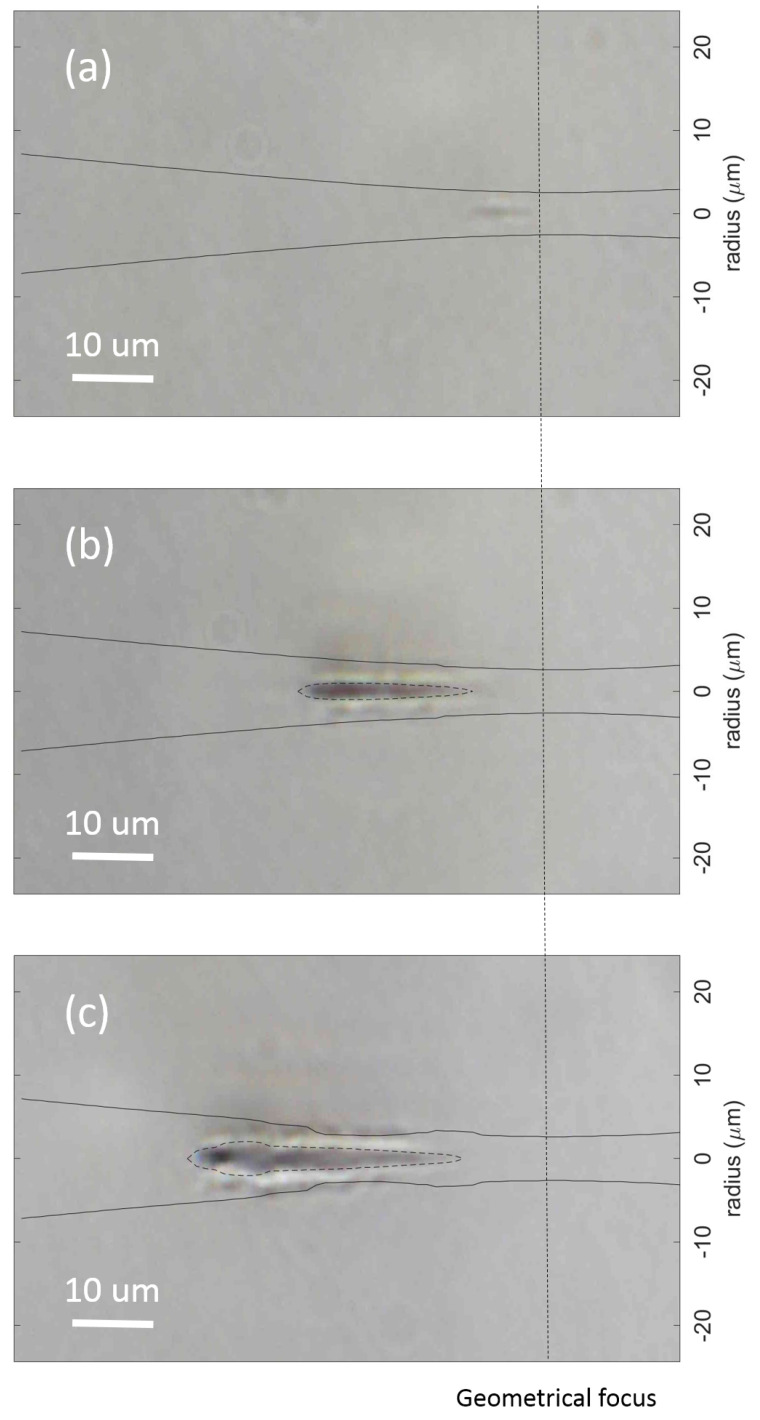
Simulation results of beam propagation and electron density overlaid on the experimental results. The input pulse energy are (**a**) 1 µJ, (**b**) 3 µJ, (**c**) 5 µJ. The solid line indicates the beam radius at $1/e^{2}$ intensity level. The dashed contour indicates the max electron density at the level of $\rho_{max}=10^{20}$ ${\mathrm{cm}}^{-3}$, and the vertical dashed line indicates the position of the geometrical focus.

The simulation results of beam propagation and electron density at input pulse energy of $E_{in}=1$ µJ, $E_{in}=3$ µJ, and $E_{in}=5$ µJ are shown in Figure 4a–c, respectively. The laser pulse is launched from the left hand side to the right hand side and the geometrical focus is marked around the vertical dashed line in the picture. At input pulse energy of 1 µJ, the material damage is barely visible and it happens around the geometrical focal position. At input pulse energy of 3 µJ, the damage track becomes obvious with smooth inner dark core and outer boundary. The inner dark core matches very well with electron density distribution at level of $\rho_{max}=10^{20}$ ${\mathrm{cm}}^{-3}$ indicated by the dashed contour shown in Figure 4b. The position of the damage track shifted towards the incoming laser beam and the beam started experiencing self-focusing at this energy level. Further increasing the input pulse energy to 5 µJ generates a longer damage track with a hollow beaded structure in the center along the propagation axis. The shape and length of the inner damage track are matched very well with electron density distribution at the level of $\rho_{max}=10^{20}$ ${\mathrm{cm}}^{-3}$ with features like the leading hollow bead structure matching the beginning portion of the electron contour and the thin tail of the damage track matching the tail of the electron contour shown in Figure 4c. The peak power of the pulse with input pulse energy of 5 µJ is $P_{peak}=0.5$ MW, which is about 7 times less than critical power $P_{cr}=3.5$ MW for self-focusing in borosilicate glass. However, the beam still experienced self-focusing and plasma defocusing as shown in the simulation results in Figure 4c, which indicates that the Kerr self focusing term in Equation (2) should not be neglected. Our simulation and experimental data are best fitted when the multiphoton ionization cross section $\sigma_{4}=4\times 10^{-41}$ ${\left({\mathrm{cm}}^{2}/W\right)}^{4}$ and the avalanche ionization coefficient $\eta =\sigma /U_{i}=1.63$ ${\mathrm{cm}}^{2}/J$.

**Figure 5:**
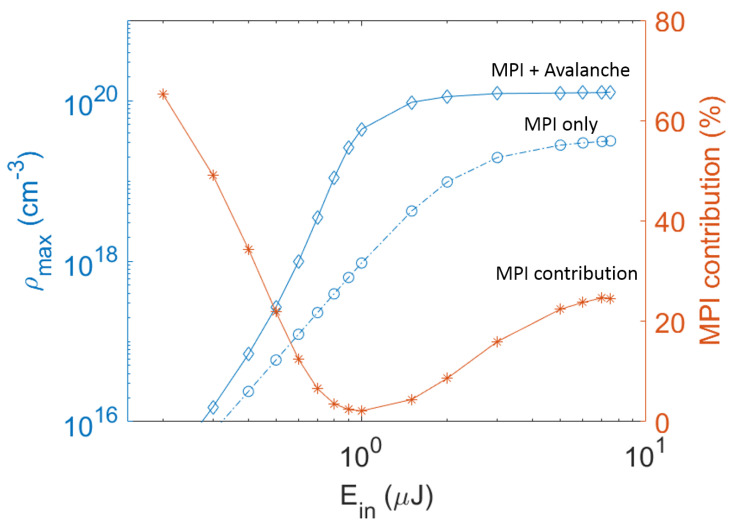
Simulation results of electron density vs. input pulse energy. The maximum electron density is indicated by the left hand side Y axis. The diamond solid line shows electron generation from multiphoton ionization and avalanche ionization. The circle dashed line shows electron generation from multiphoton ionization only. The percentage of electron contribution from multiphoton ionization is indicated by the right hand side Y axis, and plotted as starred solid line in the graph. MPI: multiphoton ionization.

The relative role of electron contribution from multiphoton ionization and avalanche ionization is shown in Figure 5. The maximum electron density clamps at around $\;10^{20}$ ${\mathrm{cm}}^{-3}$ when input pulse energy approaches 2 µJ, similar electron clamping density has been found in other studies as well [5,26]. At this energy level and above, the damage track has a distinct dark inner core as shown in Figure 2b–d, corresponding to input pulse energy of 2 µJ, 3 µJ, 5 µJ, respectively. At low energy level $E_{in}<0.2$ µJ, multiphoton ionization is the major channel for electron generation with more than 60% of the electrons generated by the multiphoton ionization process. When increasing the pulse energy, the electron contribution from multiphoton ionization decreases while the contribution from avalanche ionization increases. At energy level of about $E_{in}=1$ µJ, the role of avalanche ionization becomes dominating with more than 98% of electron contribution, while multiphoton ionization provides about 2% of the electrons. At energy level $E_{in}>1$ µJ, the percentage of electron generation from multiphoton ionization increases but the avalanche ionization still remains as the major effect with more than 75% of electron contribution.

## 5. Conclusions

In conclusion, we demonstrated generating damage tracks in the bulk of borosilicate glass by a single shot of IR picosecond laser pulse. Particularly, we generated extended damage tracks with an aspect ratio of 1:10 under our focusing geometry. The positions of the damage track are shifting away from the geometrical focus towards the incoming laser beam driven by the plasma expansions and nonlinear effect such as self-focusing. The shape of the damage track shows distinct features at different energy levels, specifically, we observed smooth damage channel at low input pulse energy levels, while beaded hollow structures were found at higher input pulse energy levels. We modeled the beam propagation and electron generation numerically. Our simulation results shows the size and shape of the inner dark damage track in the experiments match very well with electron density of $\rho_{max}=10^{20}$ ${\mathrm{cm}}^{-3}$ in our simulation. The dynamics of electron generation at different energy levels has been investigated. We found multiphoton ionization is the major channel for electron generation at low pulse energy level. The role of multiphoton ionization becomes smaller and the role of avalanche ionization becomes bigger as we increase the pulse energy. The percentage of electron contribution from avalanche ionization reaches a maximum of 98% at pulse energy of 1 µJ in our simulation. Further increasing the pulse energy to above 1 µJ leads to a bigger role of the multiphoton ionization but the avalanche ionization still remains a significant contributor. Our study shed light on the fundamental mechanism of electron generation that eventually led to material damages in the bulk of borosilicate glass. The results presented here might help to precisely control electron generation during ultrashort pulse laser processing of transparent materials in applications such as micro-welding, waveguide writing, and microfluidics.

## Abbreviations

The following abbreviations are used in this manuscript:

MPI	multiphoton ionization
E	electrical field envelop
$\omega_{0}$	carrier wave frequency
*c*	speed of light in air
$w_{0}$	beam radius
$w_{f}$	beam waist
$z_{f}$	Rayleigh length
$t_{p}$	FWHM pulse duration
*k*	wavenumber
*f*	beam curvature
$n_{2}$	nonlinear refractive index
$P_{cr}$	critical power for self focusing
σ	cross section for inverse bremsstrahlung process
$\tau_{c}$	electron collision time
$\rho_{c}$	critical plasma density
$U_{i}$	band gap
γ	Keldysh parameter
$W_{PI}$	photon ionization rate
$\sigma_{4}$	cross section for multiphoton ionization
η	avalanche ionization coefficient
$\rho_{at}$	background atom density
$\tau_{r}$	electron recombination time
